# Evaluation of a point-of-care immunoassay test kit ‘StrongStep’ for cryptococcal antigen detection

**DOI:** 10.1371/journal.pone.0190652

**Published:** 2018-01-05

**Authors:** Edward Mpoza, Liliane Mukaremera, Didas Atwebembere Kundura, Andrew Akampurira, Tonny Luggya, Kiiza Kandole Tadeo, Katelyn A. Pastick, Sarah C. Bridge, Lillian Tugume, Reuben Kiggundu, Abdu K. Musubire, Darlisha A. Williams, Conrad Muzoora, Elizabeth Nalintya, Radha Rajasingham, Joshua Rhein, David R. Boulware, David B. Meya, Mahsa Abassi

**Affiliations:** 1 Infectious Diseases Institute, Kampala, Uganda; 2 University of Minnesota, Minneapolis, Minnesota, United States of America; 3 Makerere University Johns Hopkins University Research Collaboration, Kampala, Uganda; 4 Department of Microbiology, Makerere University, Kampala, Uganda; 5 Mbarara University of Science and Technology, Mbarara, Uganda; Public Health England, UNITED KINGDOM

## Abstract

**Background:**

HIV-associated cryptococcal meningitis is the leading cause of adult meningitis in Sub-Saharan Africa, accounting for 15%–20% of AIDS-attributable mortality. The development of point-of-care assays has greatly improved the screening and diagnosis of cryptococcal disease. We evaluated a point-of-care immunoassay, StrongStep (Liming Bio, Nanjing, Jiangsu, China) lateral flow assay (LFA), for cryptococcal antigen (CrAg) detection in cerebrospinal fluid (CSF) and plasma.

**Methods:**

We retrospectively tested 143 CSF and 77 plasma samples collected from HIV-seropositive individuals with suspected meningitis from 2012–2016 in Uganda. We prospectively tested 90 plasma samples collected from HIV-seropositive individuals with CD4 cell count <100 cells/μL from 2016–2017 as part of a cryptococcal antigenemia screening program. The StrongStep CrAg was tested against a composite reference standard of positive Immy CrAg LFA (Immy, Norman, OK, USA) or CSF culture with statistical comparison by McNemar’s test.

**Results:**

StrongStep CrAg had a 98% (54/55) sensitivity and 90% (101/112) specificity in plasma (*P* = 0.009, versus reference standard). In CSF, the StrongStep CrAg had 100% (101/101) sensitivity and 98% (41/42) specificity (*P* = 0.99). Adjusting for the cryptococcal antigenemia prevalence of 9% in Uganda and average cryptococcal meningitis prevalence of 37% in Sub-Saharan Africa, the positive predictive value of the StrongStep CrAg was 50% in plasma and 96% in CSF.

**Conclusions:**

We found the StrongStep CrAg LFA to be a sensitive assay, which unfortunately lacked specificity in plasma. In lower prevalence settings, a majority of positive results from blood would be expected to be false positives.

## Introduction

HIV-associated cryptococcal meningitis is the leading cause of adult meningitis in Sub-Saharan Africa and accounts for 15%–20% of AIDS-attributable mortality[1–3]. The global prevalence of asymptomatic cryptococcal antigenemia (CrAg) in HIV-seropositive individuals averages approximately 6% with an estimated prevalence of 8.8% in Kampala, Uganda[1]. Screening and preemptive treatment of cryptococcal antigenemia has been shown to be a cost effective method of averting cases of cryptococcal meningitis. Therefore, the World Health Organization and Ugandan National Guidelines recommend screening all HIV-seropositive individuals with a CD4 cell count <100cells/μL for the presence of cryptococcal antigen, followed by preemptive fluconazole therapy[4, 5]. Despite recent advancements in diagnostic tools, CrAg screening, as well as the rapid and accurate diagnosis of cryptococcal meningitis, continues to remain a challenge due to the unavailability of point-of-care assays, interruptions in the supply chain resulting in unreliable, non-continuous screening, and lack of expertise and/or laboratory facilities for CrAg testing in resource-limited settings[6–8].

In July 2011, a lateral flow immunochromatographic assay (Immy Inc., Norman, OK, USA) was approved by the US Food and Drug Administration for detection of CrAg in CSF and plasma. The CrAg LFA is a rapid diagnostic test that provides a definitive result (positive/negative) in ≤15 minutes. In multiple validation studies, the Immy CrAg LFA has repeatedly demonstrated superior diagnostic performance with a sensitivity of ≥99% and specificity of ≥99%, outperforming other diagnostic tests [9–11]. The CrAg LFA is currently being used as the ‘gold standard’ for CrAg detection throughout Uganda.

We set out to evaluate a point-of-care immunoassay, StrongStep (Liming Bio, Nanjing, Jiangsu, China), for CrAg detection in CSF and plasma. The StrongStep LFA was tested against a composite reference standard of a positive Immy CrAg LFA or positive CSF fungal culture.

## Methods

### Study design

We evaluated the diagnostic performance of the StrongStep CrAg rapid test device by retrospectively and prospectively testing CrAg-positive and CrAg-negative CSF and plasma samples. Retrospective samples were collected from HIV-seropositive participants between 2012–2016 at Mulago Hospital in Kampala, Uganda and from Mbarara Regional Referral Hospital in Mbarara, Uganda. Specimens originated from four prospective cohorts: Cryptococcal Optimal Antiretroviral therapy Timing (COAT) trial [12], Neurocognitive Outcomes on Antiretroviral Therapy (NOAT)[13, 14], and Adjunctive Sertraline for the Treatment of HIV-associated Cryptococcal Meningitis (ASTRO-CM) [15]. We also prospectively tested plasma specimens collected from the Integration of Community-based Cryptococcal Antigen Screening into Routine HIV Care in Uganda among asymptomatic patients with CD4<100 cells/μL between September 2016-March 2017. All participants provided written informed consent to store their samples for future diagnostic studies. Ethical approval was granted from the Uganda National Council of Science and Technology, Mulago Hospital Research and Ethics Committee, Makerere University Institutional Review Board, and the University of Minnesota.

### The CrAg Immunoassay ‘StrongStep’ principle

The CrAg Immunoassay kit, StrongStep, is an immunochromatographic assay impregnated with monoclonal antibodies with the ability to detect the capsular polysaccharide antigen of *Cryptococcus neoformans* and *Cryptococcus gattii*. The StrongStep assay was performed following the manufacturer’s instructions. Briefly, two drops (80μl) of specimen were added to the LFA test well, ensuring that no air bubbles were trapped into the well. Results were read after ten minutes.

### Qualitative validation

All retrospective CSF and plasma samples tested were stored at -80°C. Frozen samples were completely thawed and kept at room temperature, for no longer than one hour, prior to testing. Prospectively, plasma samples were collected in EDTA coated vacuum containers and stored for <48 hours at 4°C prior to testing.

### In vitro analytical sensitivity

Semi-quantitative titration was performed on the StrongStep CrAg LFA using the Immy CrAg positive control (Glycine-buffered saline spiked with cryptococcal glucuronoxylomannan antigen). Dilutions were prepared with an initial dilution of 1:40, followed by two-fold serial dilutions up to 1:5120. Semi-Quantitative titration was performed simultaneously on the Immy and StrongStep LFA and repeated in triplicate with results verified by three independent readers.

### Statistical analysis

We evaluated the diagnostic performance (i.e. sensitivity, specificity, positive predictive, and negative predictive values) of the StrongStep CrAg in plasma and CSF. Each StrongStep CrAg test was compared to a composite reference standard of a positive Immy CrAg LFA or positive CSF culture for *Cryptococcus neoformans* (when available for persons with meningitis). We compared the StrongStep CrAg to the reference standard Immy CrAg LFA for all prospectively collected plasma samples routinely collected for CrAg screening program in accordance with international guidelines [16].

The positive and negative predictive values for plasma samples were calculated to adjust for the cryptococcal antigenemia prevalence of 9% in HIV-seropositive persons with CD4<100 cell/μL in Kampala, Uganda[6]. We also used a pooled average of 37% prevalence of cryptococcal meningitis in Sub-Saharan Africa to adjust for the positive and negative predictive values of a positive CSF test [17]. Adjusted positive and negative predictive values based on disease prevalence were calculated using Bayes Theorem (Positive Predictive Value = Sensitivity x prevalence/sensitivity x prevalence + (1-specificity) x (1-prevalence)). The Kappa (κ) statistic determined percent agreement. McNemar’s test assessed the statistical significance between the results obtained with the StrongStep and the composite reference standard. Data were compiled initially in Microsoft Excel 2016 and then analyzed using R version 3.2.1 (2015-June-18).

## Results

We tested a total of 310 samples (143 CSF and 167 plasma) from 282 participants (28 participants contributed both CSF and plasma samples). There were 156 confirmed cases of cryptococcal disease (101 CSF and 55 plasma) and 154 cases of non-cryptococcal disease (42 CSF and 112 plasma). Of the participants’ samples with identifiers, 112 were from men, and 124 were from women. The median age was 32 years (interquartile range (IQR), 28 to 40; max 68 years) with a median CD4 count of 29 cells/μL (IQR, 9 to 73, max 658).

Culture data were available for 142 of 143 CSF samples and 54 of 167 plasma samples tested. Of 101 persons with cryptococcal meningitis positive by the composite reference standard, 100 were CSF CrAg-positive by Immy CrAg LFA and 91 were CSF culture positive for *Cryptococcus neoformans*. Of 10 culture negative persons, all were CSF CrAg-positive and plasma CrAg-positive with a physician diagnosis of cryptococcal meningitis. One CSF and one plasma sample had negative test results by Immy CrAg LFA, but these two participants had positive CSF cultures and a clinical diagnosis of cryptococcal meningitis. The CrAg LFA is noted to be more diagnostically sensitive than quantitative CSF culture, but no test is perfect, thus a composite reference standard was utilized[9].

We found that the CrAg Immunoassay, StrongStep, had a sensitivity of 98% (54/55) and specificity of 90% (101/112; 95% confidence interval (CI), 83% to 95%) in plasma. When tested on CSF, the StrongStep had a sensitivity of 100% (101/101) and specificity of 98% (41/42; 95%CI, 87% to 99.9%) Table 1. Adjusting for the cryptococcal antigenemia prevalence of 9% in Kampala, Uganda and cryptococcal meningitis prevalence of 37% in Sub-Saharan Africa, the positive predictive value of the StrongStep CrAg was only 50% in plasma and 96% in CSF. The negative predictive value was 99.8% in plasma and 100% in CSF.

**Table 1:** Performance characteristics of the StrongStep LFA in Uganda.

Specimen	N	Sensitivity	Specificity	Adjusted Positive Predictive Value	Adjusted Negative Predictive Value	P-value
CSF	143	100% (101/101)	98% (41/42)	96%	100%	0.99
Plasma	167	98% (54/55)	90% (101/112)	50%	99.8%	0.009

Data are presented as percentage (numerator/denominator). The adjusted positive and negative predictive values are calculated for a CrAg antigenemia prevalence of 9% in Kampala, Uganda and cryptococcal meningitis prevalence of 37% in Sub-Saharan Africa [6, 17].

The overall agreement between the StrongStep CrAg LFA and the Immy CrAg LFA in CSF was 99% (κ = 0.98; 95%CI, 0.95 to1.0) with a positive percent agreement of 99.5% (95%CI, 98.5% to 100%) and negative percent agreement of 99% (95%CI, 96% to 100%). There was no statistically significant difference between the results obtained from the two tests in CSF (McNemar’s test, p = 0.99) Table 1. The overall agreement between the StrongStep CrAg LFA and the Immy CrAg LFA in plasma was 93% (κ = 0.84; 95%CI, 0.76 to 0.93) with a positive percent agreement of 90% (95%CI, 84% to 96%) and negative percent agreement of 94% (95%CI, 91% to 98%) respectively. In plasma, the StrongStep was statistically different than the composite reference standard (McNemar’s test, p = 0.009) Table 1.

The StrongStep CrAg LFA had a failure rate of 1.6% (5 failed tests out of 310 total tests performed). Failed tests resulted from the failure of the sample specimen to migrate up the test strip and produce a visible control line. The failed tests were discarded, and the sample was re-run on a new kit. None of the Immy CrAg LFA tests failed when run.

The StrongStep LFA was able to correctly identify all negative CSF samples as true negative results. However, the StrongStep LFA misclassified 1 CSF samples and 11 plasma samples as a false positive result Table 2.

**Table 2:** Characteristics of CSF and Plasma specimens misclassified by the StrongStep LFA.

Specimen	N	StrongStep CrAg LFA	Immy CrAg Plasma	Immy CrAg CSF	CSF culture	Clinical Diagnosis	Classification
CSF	1	Positive	N/A	Negative	Negative	No Meningitis	False Positive
Plasma	1	Negative	Negative	Positive	39,000 CFU/mL	Cryptococcal Meningitis	False Negative
Plasma	2	Positive	Negative	Negative	Negative	Viral Meningitis	False Positive
Plasma	9	Positive	Negative	N/A	N/A	No CrAg Antigenemia	False Positive

CSF = cerebrospinal Fluid; CrAg = cryptococcal antigen; CFU = colony forming units; LFA = lateral flow immunochromatographic assay; N = number of specimens; N/A = not available.

We also performed the semi-quantitative titration procedure on the StrongStep LFA in order to determine the assay’s degree of sensitivity in detecting cryptococcal antigen at various dilutions. The StrongStep LFA and the Immy LFA were tested simultaneously, using the Immy CrAg positive control, on three separate titration rounds and using the same dilution specimen. We found that the StrongStep assay repeatedly gave positive test results up to dilutions of 1:1280 as compared to the Immy LFA, which gave positive test results up to dilutions of 1:160 Table 3. The StrongStep LFA read as high as a dilution of 1:5120 on one replicate, while the Immy LFA read as high as 1:320 on one replicate.

**Table 3:** Semi-Quantitative titration of the StrongStep LFA as compared to the Immy LFA.

Test	1:40	1:80	1:160	1:320	1:640	1:1280	1:2560	1:5120
Immy 1	**+**	**+**	**+**	**-**				
Immy 2	**+**	**+**	**+**	**+**	**-**			
Immy 3	**+**	**+**	**+**	**-**	** **			
StrongStep 1	**+**	**+**	**+**	**+**	**+**	**+**	**-**	
StrongStep2	**+**	**+**	**+**	**+**	**+**	**+**	**-**	
StrongStep 3	**+**	**+**	**+**	**+**	**+**	**+**	**+**	**-**

Semi-Quantitative titration was performed using the Immy CrAg positive control with an initial dilution of 1:40, followed by 1:2 serial dilutions up to 1:5120.

+; positive test result.

-; negative test result.

blank; dilution not done.

## Discussion

This study evaluated the diagnostic performance of the StrongStep CrAg LFA compared with the Immy CrAg LFA, in detecting the presence of cryptococcal antigen in both CSF and plasma specimens collected from participants in Uganda. We found the StrongStep CrAg LFA to be a very sensitive test, but at the cost of poor specificity. This was most apparent in plasma where the StrongStep CrAg LFA had a sensitivity of 98% and specificity of 90% with a statistical difference compared to the composite reference standard (p = 0.009) and with the Immy CrAg LFA (P = 0.001). When adjusting for disease prevalence of 9% cryptococcal antigenemia, the positive predictive value of the StrongStep CrAg in plasma was only 50%. This means there would be equal probability of a positive test result being a false positive as a true positive. The higher StrongStep LFA analytical sensitivity came with the cost of lower specificity with frequent false positives.

The implementation of a national CrAg screening program for early diagnosis and preemptive antifungal therapy is of utmost importance given the high mortality rates associated with cryptococcal meningitis[1]. Widespread access to point of care diagnostic tools is paramount for the rapid and accurate diagnosis of cryptococcal disease, including cryptococcal antigenemia and cryptococcal meningitis. The CrAg Immunoassay, StrongStep, gives results in 10 minutes, is easy to use, needs no special laboratory equipment, does not require a diluent, and can be kept at room temperature, thus fulfilling the World Health Organization (WHO) ASSURED criteria as a point of care test [4, 18]; however, its issue with specificity in this analysis is problematic. Reliability is also a concern, with approximately 1 out of every 60 StrongStep tests failing to work.

Within Kampala, Uganda, the predictive value of the StrongStep CrAg LFA was considerably reduced when the prevalence of cryptococcal antigenemia was taken into account. As a screening test to be used nationally, there is concern that a positive test result on plasma has only a 50% probability of being correctly identified as a true positive result. In an already strained healthcare system, the high rate of false positivity, especially in plasma, would subject people to unnecessary medical therapy and overburden an already strained healthcare system.

Through this study, we were able to evaluate the diagnostic performance of the StrongStep LFA in detecting the presence of cryptococcal antigen in the CSF and plasma of HIV-seropositive individuals in Kampala and Mbarara, Uganda. The StrongStep CrAg LFA did not diagnostically perform well in plasma and would be a problematic assay if used for a nationwide screening program in Uganda. The StrongStep CrAg LFA did show promise in cryptococcal antigen detection in the CSF (where less protein and antibodies are present). Further work is needed to determine if there are interfering antibodies that could result in a false positive test result and if there are methods to improve the specificity of the StrongStep CrAg LFA assay. At present, we cannot recommend the use of this assay in plasma based on problems with specificity.

## Supporting information

S1 Dataset Dataset for the manuscript. (XLSX)
